# Prevalence of and factors associated with dilated choroidal vessels beneath the retinal pigment epithelium among the Japanese

**DOI:** 10.1038/s41598-021-90493-z

**Published:** 2021-05-28

**Authors:** Yasuki Ito, Mari Ito, Takeshi Iwase, Keiko Kataoka, Kazuhisa Yamada, Sayuri Yasuda, Hiroki Ito, Jun Takeuchi, Yuyako Nakano, Ai Fujita, Etsuyo Horiguchi, Yosuke Taki, Hiroshi Yatsuya, Hiroko Terasaki

**Affiliations:** 1 Department of Ophthalmology, Nagoya University Graduate School of Medicine, Nagoya, 466-8550 Japan; 2 Department of Public Health and Health Systems, Nagoya University Graduate School of Medicine, Nagoya, 466-8550 Japan; 3 Department of Public Health, Fujita Health University School of Medicine, Aichi, 470-1192 Japan

**Keywords:** Medical research, Population screening, Medical imaging, Public health, Retinal diseases, Risk factors

## Abstract

Pachyvessels are pathologically dilated large choroidal vessels and are associated with the pathogenesis of several pachychoroid-related disorders, including central serous chorioretinopathy. We aimed to investigate the prevalence of and risk factors for pachyvessels in the Japanese population. We included 316 participants (aged ≥ 40 years) with normal right eyes. The presence of pachyvessels (vertical diameter > 300 µm, distance to the retinal pigment epithelium < 50 µm) was determined using 6 × 6 mm macular swept-source optical coherence tomography images, and associated risk factors were investigated. Subfoveal choroidal thickness was measured, and its associated risk factors investigated. The overall prevalence of pachychoroids was 9.5%. Regression analysis showed that a younger age, shorter axial length, male sex, and smoking were significantly associated with the presence of pachyvessels (*p* = 0.047; odds ratio [OR] 0.96 per year, *p* = 0.021; OR 0.61 per 1 mm, *p* = 0.012; OR 3.08 vs. female, and *p* = 0.011; OR 3.15 vs. non-smoker, respectively) and greater choroidal thickness (*p* < 0.001, *p* < 0.001, *p* < 0.003, and *p* < 0.017, respectively). The results were consistent with other research findings which showed that pachychoroid-related disorders such as central serous chorioretinopathy were associated with younger age, male sex, shorter axial length, and smoking. Smoking may be associated with choroidal circulatory disturbance in the Japanese population.

## Introduction

Pachyvessels are large, inwardly displaced, pathologically dilated choroidal vessels. Pachyvessels can cause focal choriocapillaris thinning and are associated with the pathogenesis of central serous chorioretinopathy (CSC)^1^. In optical coherence tomography (OCT) angiography images of CSC eyes, the area of choriocapillaris flow void was found to be high^2^, and the location of the choriocapillaris flow void was associated with the distribution of the underlying choroidal vessels^3,4^. Studies suggest that pachyvessels impair choriocapillaris and may be responsible for retinal pigment epithelium (RPE) damage, resulting in CSC. Therefore, pachyvessels may be a preclinical sign of CSC, and normal eyes with pachyvessels may have a potential risk of CSC.

Baek et al. reported that the prevalence of pachyvessels was 25%, 46%, 85%, 96%, and 100% in non-neovascular age-related macular degeneration (AMD), neovascular AMD, thin choroid polypoidal choroidal vasculopathy (PCV), thick choroid PCV, and CSC eyes, respectively^5^. However, the prevalence of pachyvessels in the normal healthy population without degenerative disease has not been studied. Thus, investigation of pachyvessel prevalence will increase understanding of its pathogenesis in CSC, PCV, and AMD. Therefore, our study evaluated the prevalence of pachyvessels and associated risk factors in a normal Japanese population. In addition, the risk factors associated with increased subfoveal choroidal thickness were studied.

## Results

Of the 316 participants included, 123 were men and 193 were women, with an average age of 62.2 ± 9.7 years and age range of 40–84 years. The average axial length was 23.9 ± 1.2 mm. Of the 316 participants, 57 were smokers and 259 were non-smokers (Table 1).

**Table 1:** Characteristics of study participants.

N	316
Age (years)	62.2 ± 9.7
Sex (male/female)	123/193
Axial length (mm)	23.9 ± 1.2
Choroidal thickness (µm)	262.6 ± 93.3
Smoker/non-smoker	57/259

The overall crude prevalence of pachyvessels was 9.5% (30/316 eyes, 95% confidence interval [CI] 6.5−13.3). Age- and sex-adjusted standardized prevalence was 13.0% (95% CI 11.8−14.2). Multivariate logistic regression analyses showed that younger age, male sex, shorter axial length, and current smoking habits were significantly associated with the presence of pachyvessels (*p* = 0.047, *p* = 0.012, *p* = 0.021, and *p* = 0.011, respectively) (Table 2).

**Table 2:** Multiple logistic regression analysis to find the factors associated with risk of pachyvessels.

	Odds ratio	95% interval	*p* value
Age (years)	0.96	0.92–0.999	0.047
Sex	3.08	1.29–7.39	0.012
Axial length (mm)	0.61	0.41–0.93	0.021
Smoking	3.15	1.30–7.64	0.011

The average choroidal thickness was 262.6 ± 93.3 µm. The choroid was significantly thicker in smokers than in non-smokers (*p* = 0.017) (Table 3). Choroidal thickness in men was thicker than that in women (*p* = 0.003) (Table 4). Multivariate regression analysis showed that shorter axial length (*p* < 0.001), younger age (*p* < 0.001), male sex (*p* = 0.003) and current smoking habits (*p* = 0.017) were associated with increased choroidal thickness (Table 5). The choroidal thickness decreased by 35.5 µm (95% CI 27.7–43.3) for each 1-mm increase in axial length, and by 4.1 µm (95% CI 3.1–5.0) for every 1-year increase in age. The choroid was 29.6 µm thicker (95% CI 5.3–53.9) in smokers than in non-smokers.

**Table 3:** Age, sex, and axial length adjusted average choroidal thickness.

	Average choroidal thickness	95% CI	*p* value
Non-smoker	256.8	247.1–266.5	0.017
Smoker	277.5	254.3–300.8	

*CI* confidence interval.

**Table 4:** Age, axial length, and smoking adjusted average choroidal thickness.

	Average choroidal thickness	95% CI	*p* value
Female	251.1	239.7–262.6	0.003
Male	280.6	266.1–295.2	

*CI* confidence interval.

**Table 5:** Multiple regression analysis to identify factors associated with subfoveal choroidal thickness.

	Unstandardized β	Standardized β	95% interval	*p* value
Age, years	− 4.10	− 0.425	− 5.0 to − 3.1	< 0.001
Sex, male vs. female	29.5	0.155	10.4 to 48.7	0.003
Axial length (mm)	− 35.5	− 0.461	− 43.3 to − 27.7	< 0.001
Smoking, smoker vs. non-smoker	29.6	0.122	5.25 to 53.9	0.017

## Discussion

This study identified younger age, shorter axial length, male sex, and current smoking habits to be significantly associated with presence of pachyvessels. In an age- and sex-matched case control study, Ersoz et al. reported that risk factors for CSC were steroid use, antidepressant or anxiolytic drug use, smoking, pregnancy, and hyperopia^6^. Furthermore, in an age-matched case control study, Chatziralli et al. reported that risk factors for CSC were male sex, high educational status, high income, smoking, obstructive sleep apnea, *Helicobacter pylori* infection, type A personality and stress, steroid use, pregnancy, and hyperopia^7^. Although the designs and populations of these studies were different from those of our study, our results were consistent with these previous studies, suggesting that presence of pachyvessels may be a preclinical sign of CSC.

Pachyvessels are also associated with the pathogenesis of PCV. There are similarities in the vascular density of the large choroidal vessel layer and pachyvessel patterns between CSC and PCV^5^. The area of maximal choroidal thickness correlates spatially with the distribution of pachyvessels and with the disease focus in eyes with PCV^8^. The risk for recurrent exudative change after three injections of anti-vascular endothelial growth factor was greater in those with pachyvessels irrespective of the presence of a pachychoroid^9^. Male sex and smoking are significant risk factors for the development of PCV, similar to pachyvessels^10^. Therefore, eyes with pachyvessels may also have a higher risk of developing PCV.

Pachyvessels are also associated with AMD. Pachyvessels have been observed in 80.6%, 44.4%, and 40% of patients with pachydrusen, soft drusen, and subretinal drusenoid deposits, respectively^11^. Ng et al. reported that pachyvessels were observed in 52.1% of eyes with exudative maculopathy and that the choroidal vascularity index significantly correlated with pachyvessels^12^.Choroidal neovascularization developed in the fellow eyes of 9% of patients with unilateral PCV or aneurysmal type 1 neovascularization, and it frequently developed in areas with RPE and outer retinal abnormalities accompanied by pachyvessels^13^. Topographically, pachydrusen location correlates with the underlying pachyvessels in eyes with pachychoroid pigment epitheliopathy, CSC, PCV, and pachychoroid neovasculopathy^14^. Therefore, in addition to CSC and PCV, eyes with pachyvessels may be at increased risk of developing AMD.

Choriocapillaris flow impairments existed in eyes with pachychoroid, and their locations were correlated with those of pachyvessels^15^. The presence of pachyvessels may cause choriocapillaris flow impairment, resulting in RPE impairment, and consequent development of pachychoroid pigment epitheliopathy, CSC, or AMD. A longitudinal observational study design is needed to show whether pachyvessels are a preclinical sign of CSC, PCV, or AMD. In addition, as smoking was a factor associated with the presence of pachyvessels, smoking cessation may be recommended for those whose eyes have pachyvessels. Further studies are needed to demonstrate the efficacy of smoking cessation by observing the changes of pachyvessels and whether the incidence of CSC or PCV decreases after smoking cessation.

The mechanism of choroidal vessel dilation is unknown. As for smoking, Spaide et al. asserted that nicotine exposure might cause nitric oxide-induced abnormalities in the choroidal vessels and induce CSC^6,7^. It is difficult to speculate how choroidal vessel dilation is affected by other factors such as age, sex, and axial length. Further studies are needed to understand how these factors affect choroidal vessel dilation.

The association between smoking and choroidal thickness has been studied with varying results^16^. There was no significant association between cigarette smoking and choroidal thickness in a study assessing Indian participants^17^ and Turkish participants^18,19^. Choroidal thickness was significantly reduced in smokers compared with the control group in a study assessing Greek participants^20^, American participants (145/147 were Caucasian)^21^, and elderly French participants (average age 82.73 years). However, the effect of smoking differs depending on genetic background. Nakanishi et al. reported on the interactions between CFH 402H and cigarette smoking in patients with PCV. The risk was higher in smokers with the CFH and/or LOC387715/HTRA1 allele than in smokers without these alleles^22^. Variants of CFH, CETP, and VEGFA exhibited different association signals in East Asian individuals in contrast to European individuals^23^. Therefore, the effect of smoking differs between races. Indeed, PCV is more common in individuals of Asian descent, accounting for up to 60% of neovascular wet AMD cases in East Asia^23–25^. In Japan, approximately half of the patients with neovascular AMD also have PCV^25^. One study that investigated the factors associated with choroidal thickness in Chinese participants with a genetic background was similar to Japanese participants^26^. In their analysis, greater choroidal thickness was significantly associated with younger age, shorter axial length, male sex, and higher number of smoking pack-years, after adjusting for age and axial length. Although the association between smoking and choroidal thickness was not significant after including more independent variables, choroidal thickness still tended to be greater in smoking participants in this study. Since it is not clear whether the effect of smoking is dose dependent, their approach of considering smoking as a continuous variable rather than a binary variable may not be appropriate. Similarly, the prevalence of pachyvessels and its association with smoking may also differ depending on genetic background and race. Further studies are needed to assess whether the effect of smoking on the prevalence of pachyvessels as well as choroidal thickness differs depending on genetic and racial background.

In our study, the prevalence of pachyvessels was 9.5%. In contrast, the prevalence of pachyvessels in non-neovascular AMD, neovascular AMD, thin choroid PCV, thick choroid PCV, and CSC eyes was 25%, 46%, 85%, 96%, and 100%, respectively, in the study by Baek et al.^5^. Although the prevalence of pachyvessels in these diseases was consistent with that of normal eyes in our study, the method used to identify pachyvessels slightly differed, and, therefore, these prevalence rates cannot be directly compared. In the study by Beak et al., the presence of pachyvessels was determined by dilatation of outer choroidal vessels on an en face OCT, which corresponds to the characteristic features of large choroidal vessel dilatation and choriocapillaris attenuation on OCT raster scans. In contrast to their qualitative definition, our definition of pachyvessels was quantitative; we considered pachyvessels to have a vertical diameter of > 300 µm and a distance of < 50 µm to the RPE. To improve reproducibility, a quantitative method is better than a qualitative method, but it is unclear as to which of these methods is the best to define pathological pachyvessels. Further studies are needed to determine an appropriate method for defining pathological pachyvessels.

Swept-source-OCT (SS-OCT) was used in this study. One advantage of SS-OCT is the larger scanning depth and deeper penetration. The central wavelength of the SS-OCT used in this study was 1080 nm, and the scan depth was 3 mm. As a result, signal attenuation in the cross-sectional OCT images was much lower than in the spectral domain OCT, and better delineation of choroidal images could be obtained. Therefore, SS-OCT is advantageous for analyzing pachyvessels.

There are certain limitations to this study. The OCT angiography program used was 6 × 6 mm, and the presence of pachyvessels was only studied in the macula. A wider-area scan would have been better to evaluate the prevalence and distribution of pachyvessels. Second, as previously stated, it is not clear whether the definition of pachyvessels used in this study was the best one. Third, systemic diseases such as diabetes and hypertension were included in this study. Although the two systemic diseases do not affect choroidal thickness^26^, they might have affected the results. Fourth, diurnal variation might have affected the choroidal thickness measurement. However, since participants were randomly examined in the morning or afternoon, the effect of diurnal variation should be minimal. Further studies in which time of examination is controlled are needed. Fifth, since we did not take the intensity or duration of smoking into account in the present analysis, the sex-difference might be due to heavier intensity or longer duration of smoking in men, which needs to be confirmed in the future study. Finally, participants in this study were aged ≥ 40 years. Since a significant proportion of patients with CSC are younger than 40 years, this study is not representative of younger participants. Further studies that include younger participants are needed to confirm whether the effect of smoking is the same in younger patients.

In conclusion, the overall prevalence of pachyvessels in the normal population was 9.5%. A younger age, male sex, shorter axial length, and current smoking habits were significantly associated with the presence of pachyvessels. Similarity between the factors associated with pachyvessels in a normal population to those of CSC eyes suggests that pachyvessels may be a subclinical sign of CSC, and eyes with pachyvessels may have a higher risk of developing CSC. Smoking was associated with the prevalence of pachyvessels and increased choroidal thickness, suggesting that smoking is associated with choroidal circulatory disturbance in the normal Japanese population.

## Methods

This prospective cross-sectional study was approved by the institutional review board of Nagoya University Hospital and was conducted in accordance with the principles of the Declaration of Helsinki. Written informed consent to participate in this study was obtained from the participants before performing any procedures.

### Participants

The participants were healthy volunteers who underwent a comprehensive health examination program (Yakumo Study) in 2018, which was supported by the local government and has been conducted in the town of Yakumo in Hokkaido, Japan, since 1982. The program comprises systemic as well as ophthalmic examinations. Participants were aged ≥ 40 years.

Participants were excluded if they met any of the following criteria: (1) exhibited signs of macular disease such as age-related macular degeneration or CSC in both eyes, (2) had retinal hemorrhage suggesting retinal vein occlusion or diabetic retinopathy in both eyes, (3) had nerve fiber layer thinning in OCT suggesting glaucoma, (4) had intraocular pressure > 21 mmHg, (5) had a subfoveal choroidal thickness < 100 µm, (6) had an axial length > 28 mm, (7) had a history of steroid and anxiolytic drug use, and (8) had a SS-OCT image signal intensity < 7. Only participants who agreed to the analysis of all examination results were included in this study. The absence of macular disease was verified by spectral domain OCT (RS-3000, Nidek, Gamagori, Aichi, Japan) and color fundus photos (Canon CR-2, Tokyo, Japan). Only the right eye of each participant was examined and included in this study. Information on smoking habits was obtained from a questionnaire, and smoking status was indicated as either “smoker” for participants who were current smokers or “non-smoker” for participants who used to smoke or never smoked.

### OCT analysis

**Figure 1:**
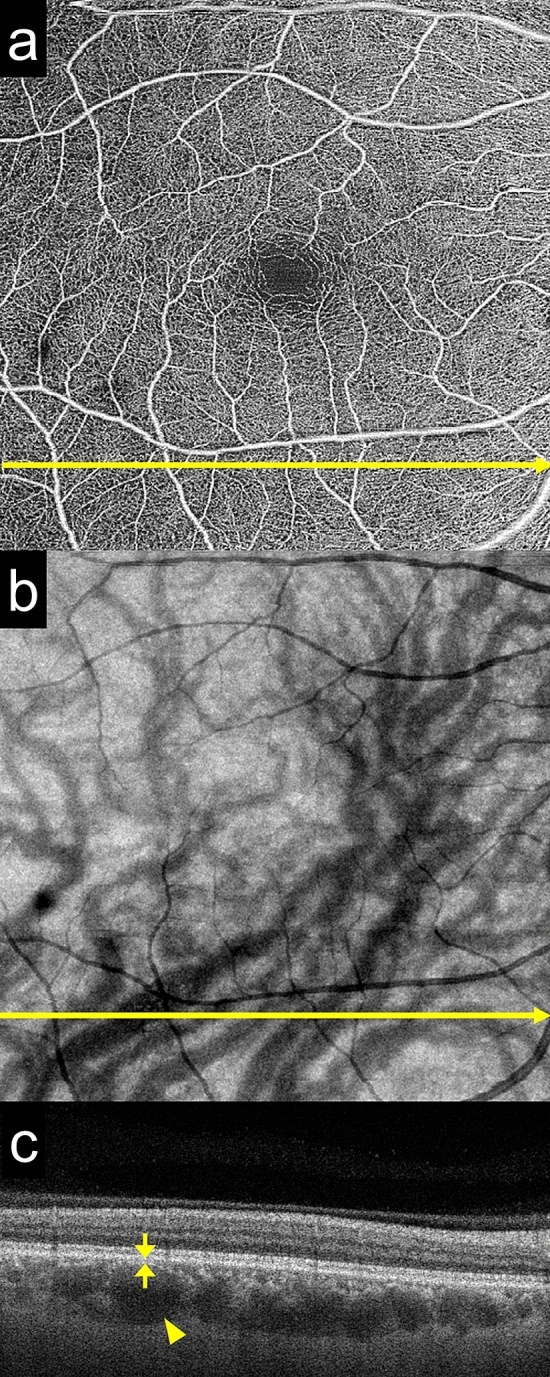
The method used to identify thick choroidal vessels (pachyvessels) near the retinal pigment epithelium (RPE). (**a**) A 6 × 6 mm swept- source optical coherence tomography (SS-OCT) image of the retinal slab. The yellow arrow indicates the location of the scan (**c**). (**b**) An SS-OCT image of the choroidal slab. A thick choroidal vessel is seen in the inferior area. The yellow arrow indicates the location of the scan (**c**). (**c**) An SS-OCT cross-sectional image taken at the location of yellow arrow in (**a**) and (**b**). Pachyvessels were identified by checking all 500 cross-sectional OCT images and en face SS-OCT images of the choroidal slab (**b**) using built-in software. Note that the thick choroidal vessel (vertical diameter > 300 µm, arrow head) is seen near the RPE, with the distance to the RPE being < 50 µm (arrow).

Pachyvessel analysis was performed using the 6 × 6 mm OCT angiography program (500 line scans with 500 A-scans) of the SS-OCT (SS-OCT, PlexElite 9000, Carl Zeiss Meditec, Dublin, CA, USA). All 500 cross-sectional OCT images and en face images of the choroid (choroidal slab) covering a 6 × 6 mm area centered on fovea in each eye were carefully evaluated by two investigators (YI and MI) to assess the presence of pachyvessels. The presence of pachyvessels was determined if a large choroidal vessel (pathologically dilated Haller vessel, vertical diameter > 300 µm^27^) was present near the RPE (distance to RPE < 50 µm) (Fig. 1). OCT angiography images were not used in this study. In the horizontal OCT images through the fovea, the subfoveal thickness was also measured using a built-in software present in the device.

### Statistical analyses

Age- and sex-adjusted standardized prevalence was calculated by the direct method using the 2015 Japan Census. Multiple logistic regression analysis was performed to identify factors associated with pachyvessels. Comparison of subfoveal choroidal thickness in the eyes of smokers and non-smokers was undertaken using analysis of covariance adjusting for age, sex, and axial length. Comparison of subfoveal choroidal thickness between men and women was also analyzed using analysis of covariance adjusting for age, axial length, and current smoking habits. Multiple linear regression analysis was performed to identify the factors associated with subfoveal choroidal thickness. For this study, a *p* value < 0.05 indicated statistical significance. IBM SPSS statistical software (version 26.0; SPSS Inc., an IBM Company, Chicago, IL, USA) was used to perform all statistical analyses.

## Data Availability

The datasets analyzed during the current study are not publicly available because of the hospital’s policy to protect personal information of the participants but are available from the corresponding author on reasonable request.
